# The Legal Position as to Medical Benefit

**Published:** 1912-09-21

**Authors:** 


The Workers' Newspaper of Administrative Medicine and Institutional Life, Administration, National Insurance and Health.

No. 1368, Vol. LII. SATURDAY, SEPTEMBER 21, 1912. PRICE ONE PENNY.

### THE LEGAL POSITION AS TO MEDICAL BENEFIT.

The weeks since July 15 are lengthening into months, and so far as outsiders can tell the disagreement between Mr. George and the medical profession concerning the adequate remuneration of the latter for attendance on persons insured under the former's Act is no nearer composure. Mr. George and Mr. Masterman seem to be so hostile to the contentions of the British Medical Association, as to be contemplating the suspension of medical benefit under the Act, substituting for it either a cash payment of six shillings per insured person or an increase in sickness benefit. We speak of the Chancellor and his satellite as contemplating such a suspension, because, although technically it will be the act of the Commissioners if it occurs, it is quite clear that it is Messrs. George and Masterman who will be the real authors and instigators of the proceeding.

Considering, therefore, that suspension of medical benefit is within measurable distance of coming to pass, it behoves the insured, the Societies, and the medical profession to scrutinise very carefully the clauses and sections of the Act which apply to this contingency. An analysis of these appears from the pen of a distinguished advocate in a contemporary,* and although the tone is that of a political opponent, the reasoning is so clear and cogent that it is worthy of careful consideration. The subject bristles with difficulties, many of which will no doubt eventually be thrashed out in the Courts, unless an amending Act succeeds in solving them. One of them is the exact construction of the all-important proviso to Section 15, which allows the Commissioners, if they are satisfied that in any district an adequate medical service cannot be obtained, to make or to authorise other arrangements for providing it or to suspend medical benefit for as long as they think fit. The clause empowers the Commissioners to pay a cash equivalent in lieu of medical benefits to insured persons, but it does not say that they must do so. Nor does it define the phrase "cash equivalent." Messrs. George and Masterman assume in their orations that six shillings will be a "cash equivalent." But it is quite open to argument whether the Courts would so adjudge it. Indeed, it is easy to conceive of circumstances in which it would be very far from being a cash equivalent. Mr. Pringle takes the case of a shepherd living five miles from the nearest doctor. No doctor can accept six shillings per annum in return for attendance (and medicine) for such an isolated worker.

Suppose, again, that immediately after January 15, when medical benefit comes into force (or is suspended), an insured person, who is informed that medical benefit is suspended, brings an action against the Commissioners for a sum of money equal to that for which he can get medical benefit on ordinary club principles. Suppose he brought to Court doctors prepared to accept him at ten shillings (or any other sum) a year, and that other doctors pronounced this to be a reasonable rate. The action could only be met, according to Mr. Pringle, by the Commissioners proving that the amount was excessive, and to do this they would have to produce doctors willing to do it at a less rate. The further question then arises whether the Courts have jurisdiction in such an action. Section 67 of the Act provides that the decision of the Insurance Commissioners is final in all disputes between an insured person and his Society or the local Insurance Committee; but it does not say that the Commissioners' decision is final where the dispute is between an insured person and themselves.

It is further shown by reference to the provisions of the Act that there is nothing therein which of necessity prevents a greater sum than six shillings per insured person being expended on medical benefits. True a very small reduction in sickness benefit might be the result, though even this is not certain. Indeed, it is almost certain that in strong Societies—that is, Societies where only good lives are accepted and where the insured are chiefly in healthy occupations—sickness benefit will eventually be much larger than as at present fixed. In

* Mr. A. S. Pringle in the *Edinburgh Medical Journal*, September 1912, p. 241.

such Societies an increase in the sum allotted to medical benefit could be made without any real risk of reducing sickness benefit. And in weaker Societies such a reduction would certainly be extremely small. There is, indeed, another alternative: the Societies may exact an extra levy from members in order to maintain sickness benefit at its initial level, if they are faced with a deficit in their accounts.

Mr. Pringle suggests that such an extra levy should be paid by all insured persons earning thirty shillings a week or more, and that it should be the sum of one penny a week. He further suggests that the medical profession, which has always been accustomed to do much work for the really poor on a charitable basis, should be asked to accept the six shillings flat rate for workers earning less than thirty shillings, receiving in return the concession of a ten shillings rate for those earning more than that sum per week. We do not suppose that either Labour leaders or the officials of approved Societies will regard this proposal with much favour; and hence it is not very likely that the Chancellor will either. Nor do we disguise from ourselves the fact that it would be difficult to work such a differential limit in the case of men or women whose wage fluctuates from week to week above and below it. Nor do we venture to predict what sort of reception the proposal would obtain from the British Medical Association. But it is quite plain that something has got to be done to clear up the muddle into which the relations between Mr. George and the medical profession have been allowed to drift; and any suggestion which seems to contain a germ of a compromise between the protagonists is worth examining, not so much in the interests of the Chancellor's vanity or the medical profession's pocket as in those of the insured workers round whom the quarrel centres. As matters stand, the lawyers seem to be the only people to whom the vaunted fruit of the Act will prove particularly refreshing.

